# Plasma Extracellular Vesicles Identify Patients With Prodromal‐ and Alzheimer's Disease Through Their Proteomic Content

**DOI:** 10.1002/alz70856_100924

**Published:** 2025-12-25

**Authors:** Maria Capdevila, Raquel Puerta, Rosanna Rossi, Laura Montrreal, Álvaro Muñoz‐Morales, Clàudia Olivé, Pamela Martino‐Adami, Laura Guzmán, Marina Carrasco, Marta Marquié, Montserrat Alegret, Antonio Camins, Alfredo Ramirez, Mercè Martí, Maria Isabel Pividori, Mercè Boada, Agustin Ruiz, Miren Ettcheto Arriola, Amanda Cano, Pablo García‐González

**Affiliations:** ^1^ Ace Alzheimer Center Barcelona, Barcelona, Spain; ^2^ Universitat Autonoma de Barcelona, Barcelona, Barcelona, Spain; ^3^ Division of Neurogenetics and Molecular Psychiatry, Department of Psychiatry and Psychotherapy, Faculty of Medicine and University Hospital Cologne, University of Cologne, Cologne, Germany; ^4^ University of Barcelona, Barcelona, Spain; ^5^ Universitat Autonoma de Barcelona, Barcelona, Please select:, Spain; ^6^ Ace Alzheimer Center Barcelona‐Universitat Internacional de Catalunya, Barcelona, Spain; ^7^ University of Texas Health Science Center, San Antonio, TX, USA; ^8^ Ace Alzheimer Center Barcelona, Barcelona, Barcelona, Spain; ^9^ Ace Alzheimer Center Barcelona – International University of Catalunya (UIC), Barcelona, Spain

## Abstract

**Background:**

Alzheimer's disease (AD) is commonly diagnosed when neuronal damage is already established and irreversible. A differential diagnosis in the mild cognitive impairment (MCI) stage is one of the greatest challenges nowadays. Blood biomarkers, and specifically plasma extracellular vesicles (pEVs), are gaining much interest as a new biomarkers’ source for the early stages of AD. This work aims to evaluate the proteomic profile of pEVs from patients with MCI and AD to explore their potential as AD screening tools.

**Methods:**

pEVs were isolated by ultracentrifugation from patients with MCI Aβ(+) (*n* = 50), MCI Aβ(‐) (*n* = 50), and with AD dementia (*n* = 43). Nanoparticle tracking analysis (NTA) and cryo‐TEM were used to characterize the pEVs. CSF, serum and pEVs proteomics were carried out by using the multiplex PEA technology of Olink^©^ proteomics, Inflammation and Neurology Explore 364 panels (728 proteins).

**Results:**

Characterization results showed that isolated plasma fraction corresponded in shape, size and concentration to EVs. pEVs’ biomarkers correlated with common AD signatures (CSF Aβ42 and pTau181, plasma pTau181, MMSE, Age and Qalb) with the same pattern than CSF biomarkers as previously described. Several neurology proteins of the pEVs (e.g. MMP‐8, MMP‐9, IGF2R or NDRG1) didn’t exhibit differences between the MCI Aβ(+) vs AD dementia groups, whilst MCI Aβ(‐) vs AD dementia did. Likewise, many pEVs neurology proteins significantly correlated (rho>0.30 and *p* <0.05) with their CSF homonyms, not instead with their serum homonyms (e.g. APP, NOS1, CXCL11, SOD2, BCAN …).

**Conclusion:**

Preliminary results suggest that the biomarkers signature of pEVs could inform about the AD pathology in the prodromal stages of AD continuum. However, further experiments are still needed for a better understanding of the EVs’ role in the AD development and pathology dissemination.

